# Isolated Cerebral Mucormycosis Caused by *Lichtheimia* Species in a Polytrauma Patient

**DOI:** 10.3390/diagnostics12020358

**Published:** 2022-01-31

**Authors:** Vasiliki Mamali, Christos Koutserimpas, Olympia Zarkotou, Georgia Vrioni, George Samonis

**Affiliations:** 1 Department of Clinical Microbiology, Tzaneio General Hospital of Piraeus, 185 36 Pireas, Greece; mamalivasiliki@outlook.com.gr (V.M.); olyzar@hotmail.com (O.Z.); 2 Department of Orthopaedics and Traumatology, 251 Hellenic Air Force General Hospital of Athens, 115 25 Athens, Greece; chrisku91@hotmail.com; 3 Department of Microbiology, Medical School, National and Kapodistrian University of Athens, 157 72 Athens, Greece; gvrioni@med.uoa.gr; 4 Department of Internal Medicine, University Hospital of Heraklion, 715 00 Heraklion, Greece

**Keywords:** fungal cerebral infection, fungal trauma, mucormycosis, fungal infection, *Mucorales*, zygomycosis

## Abstract

Isolated post-traumatic cerebral mucormycosis represents an extremely rare and severe disease. A case of isolated cerebral mucormycosis infection caused by *Lichtheimia* spp. in a 21-year-old multi-trauma patient is presented. The patient was hospitalized in the intensive care unit and underwent craniotomy due to brain injuries. Two weeks following the initial procedure, pus drained from the surgical wound was microscopically examined and cultured, yielding *Lichtheimia* spp. Imaging showed parietal, temporal and frontal abscesses at the right side. The patient was commenced on amphotericin B and underwent surgical debridement, while histopathological examination of the affected tissue demonstrated broad, aseptate hyphae, findings typical for mucormycetes. The patient passed away due to heavy traumatic injuries after 2 months. It is speculated that direct inoculation was the portal of entry for infection, and that high steroid use for 2 weeks following inoculation contributed to the severity of infection that developed. Isolated cerebral mucormycosis in immunocompetent hosts is an extremely rare, but severe disease. Diagnosis is established through direct microscopy, histopathology and/or cultures. PCR-based techniques are useful either to detect mucormycetes in tissues, especially when cultures are negative, or to accurately identify the fungi grown in cultures at the species level. A high suspicion index, especially in the necrotic lesions of traumas, is of the utmost importance for early diagnosis. Appropriate surgical debridement, as well as antifungal therapy, including amphotericin B, represents the treatment of choice.

## 1. Introduction

Mucormycosis represents a severe infection caused by the fungi of the order *Mucorales*, such as *Mucor*, *Rhizopus, Rhizomucor* or *Lichtheimia* [1,2]. The infection is usually acquired by the inhalation of sporangiospores, occasionally by the ingestion of contaminated food or traumatic inoculation. These fungi, as with *Aspergillus* species, are commonly found in the soil and high-humidity climates and can, therefore, cause primary cutaneous infection via direct inoculation [1,3]. The vast majority of these infections occur in immunocompromised hosts, especially those with diabetes mellitus, hematological malignancy, solid organ transplants and under corticosteroid therapy [2].

Isolated cerebral mucormycosis represents a rare invasive fungal infection, most commonly affecting immunocompromised patients or patients with a history of intravenous drug abuse [4]. The fungi under *Mucorales* are ubiquitous, and morphologically appear as broad, aseptate or sparsely septate ribbon-like hyphae. Eleven genera and about 27 species under *Mucorales* are associated with human infections. *Rhizopus arrhizus* is the most common agent causing mucormycosis across the globe, followed by *Lichtheimia*, *Apophysomyces*, *Rhizomucor*, *Mucor* and *Cunninghamella* species [2,4,5]. Mucormycosis is associated with angio-invasion and high mortality.

An extremely rare case of isolated cerebral mucormycosis infection caused by *Lichtheimia* spp. in a 21-year-old multi-trauma patient is presented, aiming to report infection due to a rather rare species, causing life-threating disease.

## 2. Case Presentation

A 21-year-old female, with unremarkable medical history was brought to the Emergency Department due to a motorcycle accident. Upon presentation, she was hemodynamically unstable (blood pressure: 75/30 mmHg; heart rate: 110 beats per min) and disorientated.

The patient was treated according to the Advanced Trauma Life Support guidelines (ABCs). She was intubated and transferred to the intensive care unit (ICU), while an initial computer tomography (CT) scan revealed subarachnoid hemorrhage, epidural and subdural hematoma, as well as free air in the left eye socket and multiple fractures in the frontal, parietal, temporal bone and the sinus cavity. Furthermore, she had sustained fractures of the left radius and ulna and the right humerus and radius.

Due to mydriasis, a second CT scan was performed the same day, revealing exacerbation of the hematomas and edema, as well as the displacement of the midline structures. At that point, she underwent urgent bilateral decompressive craniotomy and hematoma removal.

The patient remained in the ICU. Neuromonitoring was regularly performed during the postoperative period, while she was commenced on vancomycin and meropenem, as well as steroids for the brain edema. She continued to be hemodynamically unstable and was supported. On the 15th postoperative day, pus was drained from the surgical wound and was microscopically examined and cultured.

**Figure 1:**
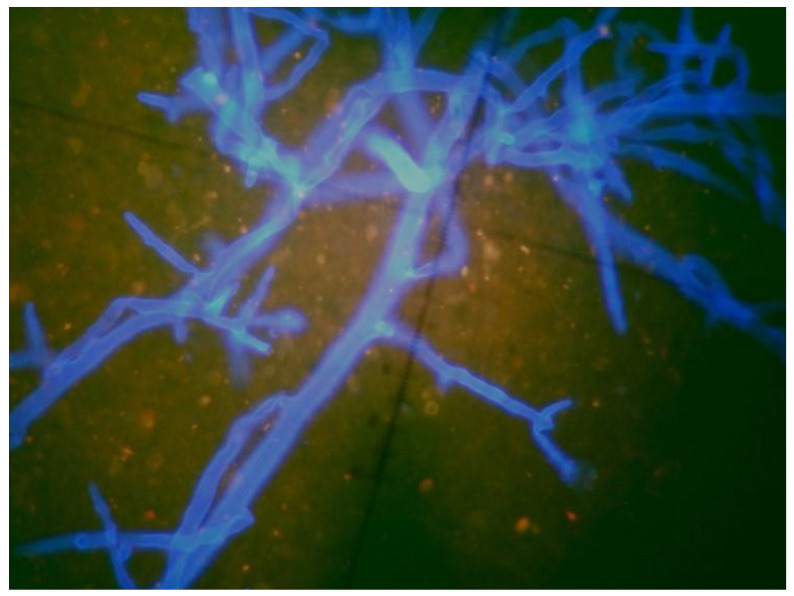
Direct microscopy with fluorescent dye (Blankophor P) revealing broad hyphae, without septa, branching at right angle, an image compatible with mucormycosis (×40 magnification).

Direct examination with a fluorescent brightener (Blankophor P) revealed wide hyphae, aseptate with right-angle branching, typical for mucormycetes (Figure 1). Hence, she was commenced on liposomal amphotericin B (5 mg/kg) (AmBisome). *Lichtheimia* spp. was yielded from the cultures, while liposomal amphotericin B was switched to 7 mg/kg due to the severity and the location of the infection.

**Figure 2:**
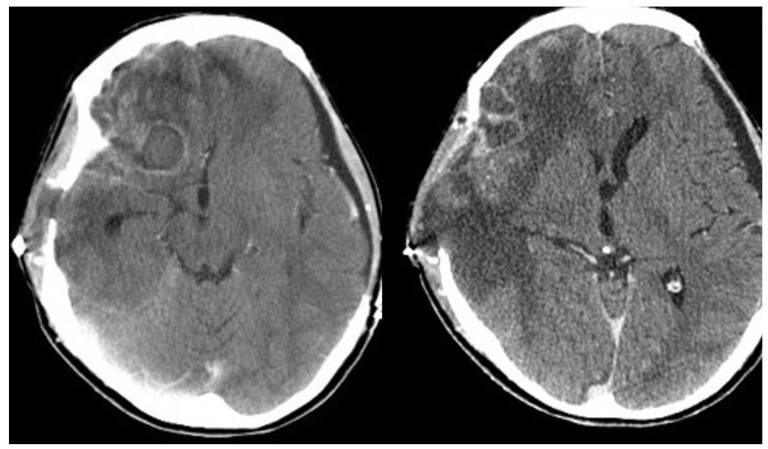
Axial contrast-enhanced CT images following bilateral craniectomy performed 2 weeks ago. The exam displayed postoperative changes more evidently in the right cerebral hemisphere, where concurrent peripherally enhancing lesions were also demonstrated, primarily suspicious of representing abscesses.

**Figure 3:**
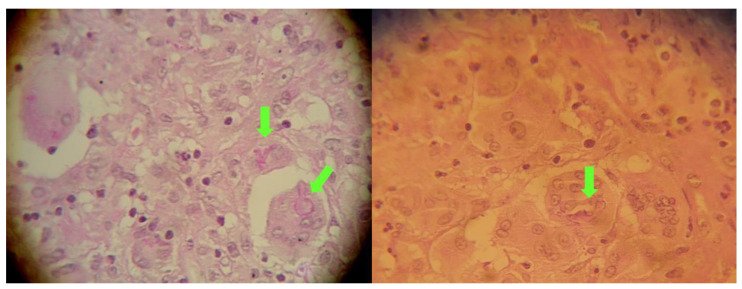
Hematoxylin and eosin (H&E) stain: ribbon-like hyphae, aseptate, branching at right angle (green arrows, magnification ×40).

**Figure 4:**
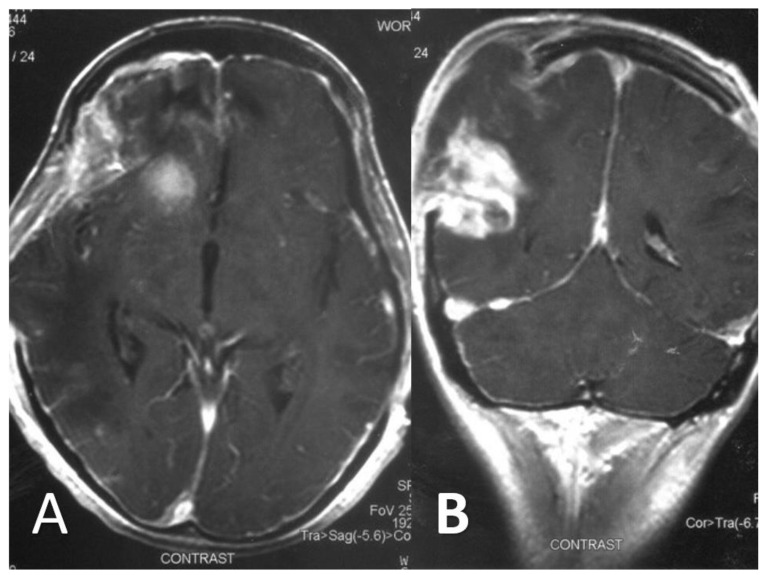
Axial (**A**) and coronal (**B**) T1 gadolinium-enhanced MR images following partial surgical debridement showcase a contrast-enhancing round lesion in the right anterior cranial fossa (**A**), and a heterogeneously enhancing lesion in the right parietal lobe (**B**). Additionally, markedly increased meningeal enhancement is also depicted in the right cerebral hemisphere (**A**), suggesting concomitant meningoencephalitis.

At that point, a new CT scan revealed parietal, temporal and frontal abscesses at the right side (Figure 2). The patient underwent surgery; surgical debridement was performed, while specimens were microscopically and histologically examined, as well as cultured. Direct microscopy with fluorescent dye (Blankophor P) again revealed the same findings, compatible with mucormycosis. Furthermore, hematoxylin–eosin stain, performed in tissue specimens, showed fungal elements similar to those with BlanKophor P (Figure 3). It is of note that cultures did not yield any fungal organism at this point, probably due to the treatment with liposomal amphotericin B. Magnetic resonance imaging (MRI) performed postoperatively, for the evaluation of the infection’s course, revealed enclosed collections absorbing contrast, indicating abscesses at the parietal, temporal and frontal lobe at the right side (Figure 4).

Following the surgical debridement, Posaconazole (300 mg per day) was added to the regimen. The patient passed away due to the heavy traumatic brain injuries 2 months after the second surgery, while on antifungal treatment. 

## 3. Discussion

Cerebral mucormycosis, is a severe infection associated with high mortality [4,6]. Diagnosis of this clinical entity is achieved through microscopy, histopathology, cultures and/or molecular testing [7]. Isolated cerebral mucormycosis in immunocompetent hosts is extremely rare. In the majority of these cases, injection drug use is present, facilitating the hematogenous spread of the mold [4]. 

The present report presented a case of isolated cerebral mucormycosis due to *Lichtheimia* spp. in a healthy female, without a history of intravenous drug use. The spread of the mold should be considered to be due to direct inoculation, making this case remarkable, while there have been only three similar cases of post-traumatic isolated cerebral mucormycosis in the literature so far [8,9,10]. 

Soil is believed to be the main habitat of most *Mucorales*, while *Lichtheimia* species can be found in farming products, such as hay and straw, as well as processed and unprocessed food products, including flour and fermented soybeans [2,6]. In global epidemiological studies, *Lichtheimia* spp. (formerly *Absidia*) seem to account for approximately 5% of all mucormycoses, However, this mold is not uncommon in cases of mucormycosis in Europe [2,6,7]. The infection occurs mainly as a result of the inhalation of asexual spores (sporangiospores) or the direct inoculation of spores into the tissue. Pulmonary infections, as well as cutaneous and subcutaneous infections, are common clinical manifestations [6]. In premature infants, this mold commonly affects the gastrointestinal tract, often resembling necrotizing enterocolitis. *Lichtheimia* species have been implicated in the form of occupational hypersensitivity pneumonitis termed farmer’s lung disease [6]. Trauma-related infections have been described in patients without obvious underlying immunosuppression, but are extremely rare [6].

The main predisposing factors for mucormycosis are hematological malignancies, organ transplantation, human immunodeficiency virus/acquired immunodeficiency syndrome (HIV/AIDS), hematopoietic stem-cell transplant, lymphoid malignancies, neutropenia, hereditary immune deficiencies, immunosuppressive medications, diabetes mellitus, intravenous drug abuse and mechanical breakdown of the blood–brain barrier through surgery or trauma [2,6,11]. This fungal infection has been associated with natural disasters and has recently been observed in India and Egypt as a post-COVID complication [12,13,14]. The reported patient had an unremarkable medical history, but suffered major trauma, leading to the breakdown of the blood–brain barrier.

Spreading through disrupted cutaneous barriers, following major trauma, represents a major route of infection in immunocompetent hosts [4,15,16,17,18]. In addition to the huge number of spores present in soil-contaminated wounds, acidosis due to large soft tissue damage and a lack of tissue viability associated with local immunodepression could explain the pathogenicity of *Mucorales* after injury [4,15,18]. It is also of note that major trauma has been shown to cause a systemic immunocompromised state [4]. Thus, necrotic lesions in poly-trauma patients should raise suspicion for such infections, since *Mucorales* may cause necrosis, restricting circulation in several vessels [6,19].

Isolated cerebral mucormycosis mainly occurs in young adults, while headache, fever, focal weakness and altered mental status represent the main symptoms. It is also of note that in most cases, the infection is localized in the basal ganglia [4,17,18]. The reported patient was also young. She had been intubated after she had been brought to the Emergency Department; hence, symptoms following the first surgical procedure and infection initiation were not evident. Nevertheless, the present case is quite different from those described so far in the literature, since the infection was due to direct inoculation, and there was no hematogenous spread. It must be noted that no other *Lichtheimia* infections had been diagnosed in the facility; hence, inoculation most probably occurred during the initial trauma and not during surgery, since the air filtration system in the operating room meets the highest standards. The infected sites in the reported patient were the right parietal, temporal and frontal lobe, as shown in the CT scan (Figure 2). 

Regarding imaging techniques indicating diagnosis, the modality of choice is brain MRI with special sequences, including gradient echo and susceptibility weighting, to assess for microhemorrhage, indicating a potentially invasive process [16]. However, as shown in the reported case, contrast CT scanning may also be useful during the diagnostic process, revealing infectious regions [4,20].

For the definite diagnosis of cerebral mucormycosis, tissue is required. The specimen should be sent for microscopy, histology and culture [7]. The characteristic findings include broad-based aseptate hyphae. In some cases, the molds do not grow in cultures, even when visible in histology [4,5,6,7]. This is mainly observed in cases in which antifungal treatment has already been administrated [6]. In the present case, the mold had grown in cultures from the initial collection of wound pus. However, the mold did not grow from samples during the second surgical procedure. Nevertheless, microscopy and histology were diagnostic on both occasions. Polymerase chain reaction (PCR) testing may be beneficial in doubtful cases [7]. However, PCR testing, in most cases, is difficult to obtain on a routine basis.

It is of note that steroid use in patients with cerebral mucormycosis has been associated with worse outcomes [4,6]. Furthermore, steroids represent an important risk for fungal infection, including mucormycosis [4,6]. Corticosteroids are commonly used for the management of edema associated with brain lesions; most commonly, methyl-prednisolone is used in high doses, dependent on the degree of brain edema [16,17]. Thus, the high suspicion of such an infection and early definite diagnosis is of the utmost importance for the avoidance of inappropriate steroid administration. Furthermore, as acidemia promotes *Mucorales* growth and binding to endothelial cells, thereby accelerating angioinvasion, the control of hyperglycemia and acidemia is critical [1,2,17].

Regarding the treatment of such infections, liposomal amphotericin B plays a pivotal role, since it has been documented that patients with isolated cerebral mucormycosis not receiving this agent had a fatal outcome [4,7]. The present patient received additional posaconazole, an agent with broad antifungal activity [21]. Hence, she received an antifungal combination that is used often in severe cases, although not encouraged by relevant guidelines [7]. Over the last decade, isavuconazole, the newest azole compound, has also been included among the treatments of choice for mucormycosis, providing an extra weapon in the antifungal armamentarium [7,22].

Isolated cerebral mucormycosis represents about 15% of cerebral mucormycosis cases, while it mainly results from an episode of fungemia [4]. The main predisposing factor in these cases, which should always be excluded from the medical history, is intravenous drug use. These infections, despite appropriate treatment, usually have a high mortality rate, exceeding 60% [2,4]. Surgical debridement represents an essential component for the successful management of this severe disease [1,7].

## 4. Conclusions

In conclusion, post-traumatic isolated mucormycosis is an extremely rare but life-threatening disease. A high suspicion index, especially in necrotic lesions in trauma patients, is pivotal for early diagnosis. Definite diagnosis through microscopy, histology and/or cultures are of paramount importance, while PCR testing may be extremely useful, especially in doubtful cases and those that antifungal treatment has already been given. Appropriate surgical debridement, as well as antifungal therapy including liposomal amphotericin B and/or isavuconazole represent the treatment of choice.

## Data Availability

Not applicable.
